# What are the views of three key stakeholder groups on extending the breast screening interval for low‐risk women? A secondary qualitative analysis

**DOI:** 10.1111/hex.13637

**Published:** 2022-10-28

**Authors:** Grace Taylor, Lorna McWilliams, Victoria G. Woof, D. Gareth Evans, David P. French

**Affiliations:** ^1^ School of Health Sciences, Manchester Centre of Health Psychology, Division of Psychology and Mental Health University of Manchester Manchester UK; ^2^ NIHR Manchester Biomedical Research Centre, Manchester Academic Health Science Centre Central Manchester University Hospitals NHS Foundation Trust Manchester UK; ^3^ The Nightingale and Prevent Breast Cancer Centre Manchester University NHS Foundation Trust Manchester UK; ^4^ Manchester Breast Centre, Manchester Cancer Research Centre University of Manchester Manchester UK; ^5^ Genomic Medicine, Division of Evolution and Genomic Sciences, St Mary's Hospital, Manchester University NHS Foundation Trust The University of Manchester Manchester UK

**Keywords:** breast cancer, interview, qualitative, risk estimation, screening, screening intervals

## Abstract

**Introduction:**

There is increasing interest in risk‐stratified breast screening, whereby the prevention and early detection offers vary by a woman's estimated risk of breast cancer. To date, more focus has been directed towards high‐risk screening pathways rather than considering women at lower risk, who may be eligible for extended screening intervals. This secondary data analysis aimed to compare the views of three key stakeholder groups on how extending screening intervals for low‐risk women should be implemented and communicated as part of a national breast screening programme.

**Methods:**

Secondary data analysis of three qualitative studies exploring the views of distinct stakeholder groups was conducted. Interviews took place with 23 low‐risk women (identified from the BC‐Predict study) and 17 national screening figures, who were involved in policy‐making and implementation. In addition, three focus groups and two interviews were conducted with 26 healthcare professionals. A multiperspective thematic analysis was conducted to identify similarities and differences between stakeholders.

**Findings:**

Three themes were produced: *Questionable assumptions about negative consequences*, highlighting how other stakeholders lack trust in how women are likely to understand extended screening intervals; *Preserving the integrity of the programme*, centring on decision‐making and maintaining a positive reputation of breast screening and *Negotiating a communication pathway* highlighting communication expectations and public campaign importance.

**Conclusions:**

A risk‐stratified screening programme should consider how best to engage women assessed as having a low risk of breast cancer to ensure mutual trust, balance the practicality of change whilst ensuring acceptability, and carefully develop multilevel inclusive communication strategies.

**Patient and Public Contribution:**

The research within this paper involved patient/public contributors throughout including study design and materials input.

## 1 BACKGROUND

The NHS breast screening programme (NHSBSP) in the United Kingdom invites women aged 50–70 years for 3‐yearly mammography screening. Since establishing the NHSBSP, a considerable dispute over the benefits and harms of screening has arisen.^1^ Harms include overdiagnosis, where a cancer diagnosis is given but if had been left undetected would not have become life‐threatening, leading to unnecessary treatment. Moreover, false‐positive test results can cause invasive testing and psychological distress, which may be sustained for up to 3 years.^2^ However, as approximately 1300 breast cancer deaths are prevented by screening each year, the Independent Panel concluded that the benefits do outweigh these harms.^1^

It has been suggested that the benefit‐to‐harm ratio of breast screening could be improved by utilizing risk‐stratified screening, whereby high‐risk women receive more frequent screening and prevention options than low‐risk women.^3^ Based on cohort studies in the United States of America, Canada, Australia and the United Kingdom, the risk of breast cancer can be accurately predicted in screening age women^4^, ^5^, ^6^, ^7^ using algorithms such as the Tyrer–Cuzick model.^8^ These models produce breast cancer risk estimates that can incorporate individual breast cancer risks based on breast density obtained from mammography, single nucleotide polymorphisms and self‐report information, such as family history, hormonal and reproductive factors. These three sources of information have been found to make an equal but distinct contribution to accurate predictions of breast cancer risk in a UK cohort study.^9^, ^10^ Although it is unclear whether this is the case for all groups of women as the participants were primarily of White ethnicity across the studies. Additionally, the models currently predict the risk of breast cancer overall, rather than by breast cancer type so it is yet unclear whether they can determine whether a woman is likely to be diagnosed with a more aggressive, less treatable tumour.

Nevertheless, risk stratification could involve high‐risk women being offered more frequent screening to identify breast cancer at an earlier stage and the uptake of risk‐reducing medication.^11^ Research has predominantly explored the potential for identifying high‐risk women, where care pathways have already been developed.^12^ The MyPeBS trial in Europe and the US‐based WISDOM trial, are both examining the effectiveness of this approach in reducing the development of stage 2+ breast cancer through earlier detection and prevention offers to women at high risk.^13^, ^14^

A neglected aspect of risk‐stratified screening concerns low‐risk women, who could experience greater harms from screening, such as overdiagnosis. Furthermore, cancers detected in women identified as low‐risk in a cohort study were more likely to be early‐stage and slow‐growing, potentially indicating that increased screening intervals would not reduce the chance of treatable breast cancer.^15^ When using the Tyrer–Cuzick model with all major predictors included, 13.5% of women received a 10‐year breast cancer risk estimate of less than 1.5%.^15^ This is equal to the mean risk of women aged 40 years who are not yet invited to the NHSBSP due to similar issues of harms, such as overdiagnosis, outweighing the benefits. This suggests that reduced screening for low‐risk women, in addition to improving the benefit‐to‐harm ratio, could further improve the cost‐effectiveness suggested for risk‐stratified screening.^16^ For example, women may be less likely to experience false‐positive test results if having fewer mammograms, which would in turn allow for the reallocation of resources to those who would benefit from more frequent screening.

Recently, the views of three key stakeholder groups in the United Kingdom have been gathered relating to the feasibility of risk stratification, with a focus on whether to reduce breast screening intervals for low‐risk women. These qualitative studies took advantage of the BC‐Predict study, which was designed to assess the feasibility of implementing risk‐stratified screening in the NHSBSP.^17^ The groups were: (a) women who had received a low‐risk estimate from the BC‐Predict study,^18^ (b) national screening figures (NSFs) involved in making and implementing decisions regarding breast screening^19^ and (c) healthcare professionals (HCPs) who were about to implement risk‐stratified screening^20^ as part of the BC‐Predict study. Interviews with low‐risk women found that they felt positive towards less frequent screening if safety evidence was available.^18^ It was concluded that communication needs careful development and should ensure informed choice. NSFs thought risk‐stratified screening would be implemented and had advantages.^19^ Yet, they were apprehensive about defining low‐risk groups, expecting a need to gain acceptability from women and demonstrate the feasibility of implementing increased intervals between screens. HCPs were concerned about less frequent screening for low‐risk women and discussed the implications of the organization of the new service.^20^

As risk‐stratified screening is likely to be implemented in some form in the near future,^3^ there is a need to establish how best to do so in a way that is acceptable to all stakeholder groups, particularly around the complex issue of communicating extended screening intervals to low‐risk women. Recommendations of how to communicate are important to ensure that risk‐stratified screening is successful in evading undermining trust in health services and to ensure that women understand the rationale behind reducing screening is to maximize their personal benefits and reduce risks.^21^ The present study, therefore, had the central aim of understanding: what are the views of key stakeholders on extending the breast screening interval for low‐risk women in relation to how this should be communicated to women.

## 2 METHODS

### 2.1 Design

A multiperspective design was implemented using qualitative secondary data attained from three primary qualitative studies which aimed to elicit the views of key stakeholders regarding the development of new care pathways for women at low risk of breast cancer. Semistructured interviews were conducted with (a) low‐risk women^18^ and (b) national healthcare policy decision‐makers, also known as NSFs.^19^ Focus groups and interviews were conducted with (c) HCPs who implemented risk‐stratified screening research.^20^ This multiperspective analysis specifically investigated differences and similarities in how these different groups perceived extended breast screening intervals for women at low risk of breast cancer.

### 2.2 Participants

Eligibility for (a) consisted of women who took part in BC‐Predict and received a low‐risk estimate, defined for this study as <2% 10‐year risk.^17^ They were identified purposively by the BC‐Predict study team with the aim to recruit a diverse sample based on ethnicity and socioeconomic background. Study invitation letters and participant information sheets (PIS) were sent by post. Eligibility for (b) NSFs included professionals with a significant profile in the NHSBSP, such as heads of the UK National Screening Committee or NICE Clinical Guidelines Committee. NSFs were recruited via purposive sampling and identified through publicly available author lists relevant to screening and invited to participate by email. Additionally, (c) HCPs who were currently working within the NHSBSP (except for general practitioners [GPs]) in Greater Manchester, East Lancashire and East Cheshire were eligible. HCPs were invited through Breast Screening Office Managers who shared information with their staff physically and via email. Invite letters and PISs were posted to GP practices in the same locations as the aforementioned breast screening sites. Contrary to previous research, HCPs in this study (aside from the GPs) worked at sites where they would be implementing risk‐stratified screening for women as part of the BC‐Predict study.

### 2.3 Procedure and materials of primary studies

The original studies were approved by Southwest Frenchay Research Ethics Committee (ref: 18/SW/0260). To investigate views on increased screening intervals for low‐risk women in the NHSBSP, interview schedules were created for the three groups (see Supporting Information: Appendices 1–3). For (a) low‐risk women^18^ and (c) HCPs^20^ the focus group/interview schedules concentrated on acceptability and feasibility. This included what information women needed to make an informed decision about having less frequent screening, how women should receive this information and how women should be supported. Women were also asked about their experience of the BC‐Predict study, however, these data were not analysed in the present study. The (b) NSFs^19^ interview schedule focused on identifying views on key implementation criteria such as risk threshold, interval length, decision‐making and implications of risk‐stratified screening. All interview schedules were informed by feedback from the relevant stakeholders, for example, medical oncologists with a national profile reviewed the NSFs schedule and covered communication issues relevant to the present study.

### 2.4 Data analysis

#### 2.4.1 Primary data analysis

The original data forming this secondary analysis were analysed using thematic analysis. Details of how each analysis was carried out can be found in the corresponding papers.^18^, ^19^, ^20^

#### 2.4.2 Secondary data analysis

Thematic analysis^22^ was conducted using principles of framework analysis to organize the data. Thematic analysis is an analytical method for systematically identifying, categorizing and proposing insight into systems of meaning across a data set.^23^ This involved the steps of familiarization, coding, developing a working analytical framework, applying the analytical framework, charting data into the framework matrix and interpreting the data.^24^ Framework was applied as it allowed the author to systematically deduce and identify summarized data from the large secondary qualitative data set that was relevant to the research question.^25^

Codes were generated inductively at a manifest level. Coding was iterative based on discussions with the analysis team, (G. T., D. P. F., L. M.). Initial codes were generated from all three stakeholder groups' data concurrently and collated into a working analytical framework of potential themes and subthemes. A multiperspective approach was used to recognize patterns in the data and identify links and contrasts between the views of key stakeholders from the three primary studies. Codes were therefore generated across all transcripts concurrently and collated. This was a suitable approach to thematic analysis as it allowed developing themes to be reflected on when applying the coding to subsequent participants.^25^ The analytical framework was applied by indexing subsequent transcripts using the existing categories and codes. The data were then charted into the framework matrix to enable thematic analysis. The framework matrix‐assisted interpretation of the data and facilitated multiperspectives by recognizing patterns and identifying links and comparisons between the views of the stakeholders on extending low‐risk pathways.^24^

Initial themes were produced by a female student studying for a Psychology Master's degree, alongside a supervisory team who discussed initial ideas and checked the themes against the data in the original papers. Also, latent themes were generated, involving a deeper level of analysis regarding how the three stakeholder groups construed and conceptualized similar issues in different ways.^26^ The final thematic structure was agreed upon with the two researchers who conducted the primary data collection (V. G. W. and L. M.).

## 3 RESULTS

### 3.1 Sample

Twenty‐three women participated in individual interviews, 20 face‐to‐face and 3 over the telephone.^18^ Interviews with 17 NSFs were conducted face‐to‐face (*n* = 10) or via telephone (*n* = 7).^19^ Three focus groups with HCPs (*n* = 26) were held face‐to‐face. Two telephone interviews were arranged for those who could not attend the focus groups.^20^ Table 1 shows demographic information about the samples.

**Table 1:** Sample characteristics of participants in the three primary studies

Characteristics of low‐risk women sample	Number
Age (years)	
46–54	16
55–64	3
65–74	4
Ethnicity	
White British or European	20
Asian or Asian British: Indian	1
Black or Black British: African	1
Mixed: White and Black African	1
Education	
A levels or equivalent	2
University degree/diploma, e.g., nursing, teaching	13
Postgraduate certificate/diploma/degree	7
Index of multiple deprivation decile	
1–2 (most deprived)	3
3–4	5
5–6	7
7–8	6
9–10 (least deprived)	2
Professional role of NSFs sample	
Breast cancer healthcare, e.g., radiology	6
Academic, e.g., health economics	6
Screening programme operations/management	5
Professional role of HCPs sample	
Mammographer/trainee mammographer/radiographer (or advanced)	15
Screening management (programme, breast imaging, screening office)	3
Cancer screening improvement lead	2
Consultant radiologist	3
General practitioner	3
Breast care nurse	1
Admin and data clerk	1

Abbreviations: HCP, healthcare professional; NSF, national screening figure.

Three themes were produced in this analysis: (1) Questionable assumptions about negative consequences, (2) Preserving the integrity of the programme and (3) Negotiating a communication pathway. Quotes are presented with participants as NSF for NSFs, a pseudonym for each low‐risk woman and the occupation of the HCP.

### 3.2 Theme 1: Questionable assumptions about negative consequences

HCPs and NSFs identified that there is a strongly held belief among those who attend the programme that screening safeguards against a diagnosis and that breast screening detect all breast cancers. There was concern that a change to the screening programme would cause apprehension amongst those at low risk regarding the frequency of monitoring, reducing the perceived protective status of the service. How women would perceive a low‐risk result was also debated, with both HCPs and NSFs concerned that an inability to understand personal risk may cause undue concern:

Radiographer 5: I think the biggest problem is that patients don't understand what risk means, and to some low risk might mean more than what they had before, because they might presume that they have no risk but they will come for screening just because that's what you do, but actually being told low risk all of a sudden you think oh actually there is a chance I may get it. (FG1)

However, women who had received a low‐risk estimate appeared accepting of the result and did not express beliefs of increased susceptibility to breast cancer or that screening prevents them from developing cancer, only that mammograms are an effective ‘safety net’ (Rebecca). However, concerns regarding women's views on effective monitoring were confirmed. Both NSFs and HCPs reported being ‘worried about how predictive the models are’ (NSF14). Similar thoughts were expressed by some women themselves, where an extended screening interval was discussed as potentially reducing the chances of detecting cancer quickly:

…if you find something early on, it's usually easier to cure or contain than it is a year down the line or six months down the line. (Elizabeth)

Yet equally, HCPs felt concerned that less frequent screening, if specifically based on informing women about screening harms, would potentially deter women from screening altogether. HCPs believed this information coupled with reduced screening intervals could falsely reassure women into thinking that they have no risk and consequently reduce breast awareness or uptake. Moreover, it could give those with poor screening attendance additional reasons to disengage with the service:

Radiographer 2: Yeah, so screening uptake would fall, just to the fact that they were told, well, you're low‐risk, so we won't bother going at all…

However, contradicting many concerns and alleviating the fears of HCPs and NSFs, low‐risk women appeared to be aware of their personal responsibilities. Many expressed understanding that low‐risk did not mean no risk and reported having a duty to be breast aware:

I would still do self‐examination despite the fact that I'm deemed to be low risk as well because low risk isn't no risk. (Lydia)

Similarly, if an extended screening interval were to be introduced, women had expectations about what information would be provided during the period in‐between mammograms, indicating that screening attendance and information about breast health would still be important despite notification of a low‐risk estimate.

### 3.3 Theme 2: Preserving the integrity of the programme

Acknowledging that the current breast screening programme is not perfect, many NSFs valued transparency within their role to convey balanced information, including that no screening is ‘a hundred percent’ (NSF7), to ensure that informed choice is promoted. However, not only do these views contrast with most HCPs who viewed the portrayal of screening harms as a deterrent against women attending screening and risks reputation of the programme, many low‐risk women viewed the harms of screening as justifiable. Experiencing possible harms was regarded as a worthwhile possibility to receive a clean bill of health and women did not find this information valuable when considering the idea to have less frequent screening:

…had I had a letter back saying ‘we've looked at your mammogram and we've detected something, would you like to come back and have it done again?’, I would have said ‘hell yes’. So even though that might be a rather stressful time … I'd rather play safe…. (Violet)

Furthermore, the practicality and feasibility of ensuring informed choice in promoting acceptability were debated. Many NSFs considered whether allowing women the option to change their screening pathway would be a more ethical approach. Yet, the expectation that women would choose to stay on a 3‐yearly screening pathway meant that this choice would become impractical and defeat the purpose of the change:

…if that was the case and the choice was offered of the sort of routine three‐yearly, or you can go along with what we've offered you, that might make it all fall down … if too many women opted to have the routine. (NSF7)

Meanwhile, many women acknowledged that taking responsibility for choosing whether to have extended screening intervals was difficult and that a programme which made decisions on their behalf would be reassuring. This opposed professionals' concerns that removing the option to remain on the current interval would limit informed choice and reduce the acceptability of the programme. When considering less frequent screening, many women reported they would stay on the existing pathway, yet without this offer, many expressed reassurance if told their screening interval was extended:

I would be happy with the medical professional making that choice. Yes. Definitely … to be honest, if it was purely my choice, I'd probably go for the additional screenings. (Marie)

For the few women who preferred having a choice about reducing their screening pathway, emphasis was placed on maintaining confidence in research and advice:

I'd rather make that decision based on more information … otherwise I would, even with the discomfort … go for every three years, for safety. (Caroline)

### 3.4 Theme 3: Negotiating a communication pathway

Most HCPs were concerned about the complexity involved in discussing breast cancer risk and confidently supporting women, especially if they are to make decisions about having less frequent screening. In particular, some identified limited capacity to have such conversations with women, with current service demands and targets taking priority over the time required to undertake training:

Radiographer 2: Not enough rooms have we, not enough staff…

Radiographer 6: …then if you're opening up conversations where we need more time and we've only got six minutes in the van to be screening ladies and then…

Consultant Radiologist 1: …They're going come with more questions aren't they? (FG1)

Relatedly, many NSFs recognized the importance of GPs for educating and reassuring women, yet reported worries related specifically to GPs' capabilities for doing so. A need for training was therefore emphasized. Similarly, some were concerned about whether pro‐screening professionals including GPs would accept an extended screening interval for low‐risk women:

The big thing about healthcare professionals is, are they going to be asked to explain it? If you've got quite a complicated screening programme … the doctor may not be an expert … that's an issue … if you're going to do something that might stimulate queries to primary care, you've got to have primary care primed. (NSF2)

…there may be other GPs who think breast screening is extremely important and would be resistant to their patients being denied the treatment. (NSF4)

Ultimately, NSFs suggested that GPs were the most appropriate group to advise women, describing them as a trusted ‘port of call’ (NSF12). However, many women reported placing greater trust in HCPs with expertise, believing that GPs would lack knowledge about risk‐stratified breast screening and felt that GP appointments for advice were not ‘urgent’ (Maxine):

Or there is an alternative person that has that more relevant information. Because no, my GP might not be the best person to go to… (Enriquetta)

All stakeholders discussed the importance of public health in the promotion of extended screening intervals for low‐risk women. Many NSFs and HCPs believed that nationwide campaigns promoting the acceptability of the programme were crucial, where some highlighted that providing information about screening harms to women has potentially damaged trust and reputation of the programme. HCPs also discussed how media campaigns could focus the message around healthcare becoming more individualized, whilst women also acknowledged the benefits of such communication approaches as a reminder that they remain in the programme in‐between screening intervals:

Mammographer 11: think that particular point, the personalisation that could be a key driver to how the women engaged, that it's going to be personal to you… (FG2)

I suppose knowing that you're in the system by getting healthy eating advice or getting advice on self‐checking, at least you know … you've not been forgotten about maybe. (Maxine)

## 4 DISCUSSION

These findings indicate the diversity of perspectives between the three significant stakeholder groups, highlighting the importance of considering the combined views of each when considering a substantial change to a national health programme. The findings suggest that the stakeholder groups have opposing views on how other groups will interpret such a change, which is likely to be detrimental to the acceptability of extending the screening interval for low‐risk women. An incongruence between the concerns of the stakeholders was identified where HCPs and NSFs displayed heightened worries that women would not view a low‐risk pathway positively. However, women raised fewer issues with the proposal and indicated that with good communication from professional experts, most of their concerns would be alleviated and that they would be confident in what the programme advised. Additionally, NSFs were concerned about the feasibility of guaranteeing efforts to ensure informed choice is maintained. Therefore, offering women the choice to have less frequent screening was viewed as more ethical, including giving them all the information available, notably the harms. However, women generally felt that the responsibility of decision‐making was difficult and felt reassured being told their screening interval was extended.

### 4.1 What this study adds to the existing literature

The previous primary research found how key stakeholders viewed the feasibility of risk‐stratified screening, in terms of whether it is acceptable. The current paper developed these findings further by highlighting how increasing screening intervals for low‐risk women should be implemented, including how it should be communicated to women. Previous studies include samples of women considering risk‐stratified screening hypothetically^27^, ^28^, ^29^ whilst the current study included women who had received a low‐risk of breast cancer allowing a more realistic view of extended screening intervals to be sought. Furthermore, the multiperspective analysis allowed an understanding of how key stakeholders construed similar issues on this topic in different ways. For example, the three stakeholder groups raised concerns of trust in each other that derived from their understanding of the proposed change, such as women would construe low‐risk as no risk. Such findings were fundamental to identifying if any aspect of the communication of an extended screening interval was unacceptable to the key stakeholders as this would challenge the feasibility of this change.

Findings revealed doubt about the role GPs would have in this aspect of risk‐stratified screening, from both NSFs and women. The NSFs highlighted the importance of GPs in supporting women, yet questioned their capability of doing so based on a lack of knowledge and evidence of acceptability. Subsequently, a need for training in primary care was emphasized. However, women appeared uneasy in receiving advice about breast cancer risk and extended screening intervals from GPs and placed more trust in HCPs with expertise. This is similar to the evidence in samples of women who desire advice from specialist HCPs to discuss being at higher risk of breast cancer.^30^ Supporting this, Spanish HCPs^31^ identified that a strong facilitator when implementing risk‐stratified screening was when information is communicated by professionals that women trust. This study also identified that implementation of risk‐stratified screening requires HCP acceptability and highlighted the importance of training and education of professionals for effective communication. The need for training and education in primary care has also been highlighted in a recent systematic review although there appears to be a lack of specific research focus on women at low risk of breast cancer.^32^

In addition, NSFs and HCPs lacked trust in women to understand the changing programme, and the current study findings showed that many women did not particularly consider the harms of screening when deciding to attend mammograms. These findings are consistent with research that demonstrates that although most women adhere to screening guidelines, they generally do not know how they were developed and that there are screening harms.^33^ Furthermore, research has shown that women require prompting to consider screening harms in low‐risk pathway scenarios, and so, addressing awareness and understanding of the harms is necessary for communicating low‐risk pathways to women.^28^

Despite heightened concerns from the professional stakeholders that women would not receive the change to low‐risk pathways well, women in fact had concerns that were less severe than both the HCPs and NSFs. Women showed that with good communication from professional experts, most worries would be alleviated, and given it is a Government programme, they would be confident in what was advised. This incongruence of views is consistent with previous research which highlighted HCP concerns about women being resistant to low‐risk pathways.^31^ Furthermore, recent qualitative research found that women overall demonstrated high acceptability towards risk‐stratified breast screening, however, were less accepting of a low‐risk pathway.^28^ An Australian study found that women were reluctant to reduce screening due to uncertainty and insecurity that low risk still means there is some risk.^34^ Additionally, participant characteristics such as lower educational level was shown to exhibit less favourable views of low‐risk pathways.^35^ Given that over 80% of participants in the present study included a sample of White British women, it is therefore uncertain what a wider group of women may think about implementing low‐risk pathways. It may be less acceptable to those not willing to take part in the study and women from ethnic minority backgrounds.

Protecting the integrity of the programme was found to be essential to effectively communicate any screening changes. The NSFs identified with this in terms of ethically supporting women and wanting to ensure that women are able to make informed choices to extend their screening interval whilst recognizing this may not be practically feasible. However, most women generally felt they would be more reassured if they were told their screening interval was extended, as the responsibility to make health‐related decisions for themselves would be too difficult. There is limited research of views on providing women agency in making their own informed health‐related decisions. However, in line with the current findings, a study of HCPs showed that 70% of participants believed that women making health‐related decisions in relation to risk‐stratified screening was important.^31^

### 4.2 Implications for practice

There are several implications for practice. Acknowledging that the key stakeholder groups appear to have different ideas of what the other groups think about and are likely to perceive an extended screening interval, which could result in a lack of trust. This lack of cohesion needs to be clarified and ideally resolved before implementation of such a change. All three stakeholder groups in the current study thought that public health campaigns could be effective in conveying information about extending the screening interval and improving understanding of the programme. However, campaigns based solely on facts may not be received well if it does not consider how to engage people in a discussion and ensure confidence between stakeholder groups within the screening programme. Similarly, tailored messages will likely be required for different groups of women.^28^

The practicalities of extending risk screening intervals need to be considered. Regardless of whether risk evidence is improved, the professional stakeholders lacked confidence in their ability to provide support to women due to their understanding that the limited time available at work would make adequate training unfeasible. Decision‐makers could consider identifying the minimum knowledge required for screening professionals and utilize those with greater risk expertise given that women are also identified as desiring this type of support. This is especially important given that the screening HCPs appeared more resistant to the change than the low‐risk women in the present study. Additionally, low‐risk pathways need to ensure that they are acceptable, yet practical, in terms of the availability of low‐risk women have to make decisions. The present research suggests that low‐risk women found decisions about their health being made for them by HCPs reassuring. However, it is important to note that it is uncertain what a wider group of women, who were not willing to take part in this study, would think about this, in addition to the NSFs in this study being concerned that limited choice for women would result in scrutiny of the programme.

### 4.3 Implications for research

Before extending the screening interval for low‐risk women, and to mitigate scrutiny, it will be necessary to provide the key stakeholders with better evidence this it is safe. Despite the Tyrer–Cuzick model being accurate at predicting low risk of cancer, future research should produce evidence regarding how many interval cancers are diagnosed in low‐risk women and at what stage they are. Such evidence could advance this model to discriminate between women who will or will not develop cancer.^36^ This would benefit risk‐stratified screening because if low‐risk results can guarantee no interval cancers between screens, it will minimize concerns.^3^

Further research with key stakeholders is also needed whereby a decisive low‐risk pathway is developed as opposed to being asked about hypothetical concepts. For example, a low‐risk pathway could be proposed that decides the number of years between screens and takes into consideration the current study's findings, such as information and reminders women would receive and who would provide support. This, in addition to including a more diverse sample of low‐risk women like recent research with British Pakistani women^37^ would further confirm the acceptability of extending the screening pathway for low‐risk women. This body of evidence will be useful for decision‐makers when considering whether to implement extended screening intervals.

### 4.4 Strengths and weaknesses

Whilst familiarizing with the original data transcripts and analyses, the first author (G. T.) identified that the original analyses (see McWilliams and colleagues^18^, ^19^, ^20^) did not consider in any depth the data containing extensive material on alternative perspectives regarding issues relevant to how low‐risk pathways should be communicated to women. Thus, the present analysis was worthwhile with these data. A particular strength of this study includes the variety of NSFs and HCPs involved. This included members of the UK National Screening Committee and HCPs currently implementing risk‐stratified screening within a feasibility study, including GPs.^19^, ^20^ This variation of roles enabled a thorough understanding of the issues based on multiple experiences. However, as women were willing to take part in the BC‐Predict study, they may have been particularly interested in breast cancer risk and breast screening. Also, over 80% of the low‐risk women sample were of White British background.^18^ It is, therefore, uncertain what a wider group of women may think about implementing low‐risk pathways.

### 4.5 Conclusions

This study has identified that key stakeholders conceptualize similar issues differently when considering communication for a risk‐stratified breast screening service which includes an extended screening interval for low‐risk women. These issues centred on professional stakeholders appearing to underestimate how women who encounter this service will react, as well as the need to protect the integrity of the programme using carefully developed communication strategies. Therefore, before implementation, a resolution of communication differences between key stakeholders is needed. Future research should continue to access multistakeholder perspectives to work towards a resolution of opinion.

## AUTHOR CONTRIBUTIONS

David P. French and D. Gareth Evans conceived and designed the primary study, data from which were re‐analysed for the present paper. Grace Taylor developed the idea for this secondary analysis, under the supervision of David P. French and Lorna McWilliams. Lorna McWilliams, David P. French and Victoria G. Woof designed the study materials. Lorna McWilliams and Victoria G. Woof identified and recruited all participants with support from D. Gareth Evans, and collected the data. Grace Taylor conducted the analysis, with input from David P. French, Lorna McWilliams and Victoria G. Woof. Grace Taylor wrote the manuscript. David P. French, Lorna McWilliams, Victoria G. Woof and D. Gareth Evans provided feedback on versions of the manuscript. All authors read and approved the final version of this manuscript.

## CONFLICT OF INTEREST

The authors declare no conflict of interest.

## ETHICS STATEMENT

Ethical approval was received from South West—Frenchay Research Ethics Committee (18/SQ/0260); all participants provided written, informed consent before taking part in an interview. Participants consented to the use of anonymized quotes in publications.

## Supporting information

Supporting information.

## Data Availability

The data that support the findings of this study are available on request from the corresponding author. The data are not publicly available due to privacy or ethical restrictions.
