# Supplementary material for: What are the views of three key stakeholder groups on extending the breast screening interval for low‐risk women? A secondary qualitative analysis
Source: Health Expect. 2022 Oct 28;25(6):3287–96. doi: 10.1111/hex.13637 (PMC9700144; doi:10.1111/hex.13637)
Supplement: Supplementary file 1 — Supporting information. [file HEX-25--s001.docx]

**Appendix 1: interview schedule (low risk women)**

*For ethics committee and researchers: this study is about exploring the views of women who have received a low risk estimate of developing breast cancer regarding increasing the screening interval beyond 3 years. The interviews will focus on, (1) what do low risk women think of an increased screening interval, (2) what information is needed to make a decision about this type of screening, (3) how should information about this increasing the screening interval be delivered, (4) what emotional effect could reducing the frequency at which women attend for screening have on women and (5) how women with a low risk should be supported by the NHS. Throughout, the interviews will focus on what is acceptable to women and how they view this proposal. We are also interested in how women would make a decision about whether to opt for reducing the frequency at which they attend for screening or not.*

**Introduction to topic**

Thank you for agreeing to talk to me today. This study is an extension to the one you took part in where you received your risk of developing breast cancer. As you received a below-average risk result we are particularly interested in talking to you. Just to refresh your memory the study you took part in was mainly focused on identifying women at high risk so they can be given the option to have more frequent screenings or medication to reduce their likelihood of developing the disease. There is some evidence that below-average risk women benefit less from screening and actually encounter more negatives. These include false positives (a mammogram that may look abnormal but after further explorative investigation is normal) and treatments which are not necessarily needed. So for women at low risk being screened less often could reduce these negatives.

What I’d like to do today is ask you what you think about the possibility of increasing the screening interval for women at below-average risk like yourself. There are no right or wrong answers we are purely interested in what you have to say. As you know we are going to be recording the interview so that I am able to concentrate on what you are saying instead of writing lots of notes. I may at times ask you to clarify what you mean and expand on things, this is not because you are not being clear it is just to make sure what you are saying is coming from you and not from me. The interview may take up to an hour depending on how much you have to say and contribute. You can stop the interview at any point if you feel you need to and you don’t need to give a reason why. Do you have any questions for me before we start?

**Interview questions and discussion points**

1. What do you remember about taking part in the BC-Predict study? (NB: may not remember it being named BC-Predict).
   1. What was your overall experience of BC-Predict? (NB: may not remember it being named BC-Predict so: what was your overall experience of the study you took part in where you received your risk?)
   2. If you can recall can you tell me a little bit about the risk letter you received?
      1. What did it tell you?
   3. What do you understand about what that risk means to you?
   4. How did you feel receiving a letter that said you are below-average risk?
   5. How do you feel about being below-average risk now?
2. How breast aware were you prior to receiving your low risk result?
   1. What does a below-average risk result mean to you with regards breast awareness now (prompt: checking breast regularly).
3. Research and healthcare professionals are suggesting that it could be safe to have screening less for women at below-average risk like you. What would you think about going for your next screen at 6 years? (So going to screening every 6 years).
   1. What concerns do you think you or women like yourself who are at below-average risk would have?
   2. What do you think are the positives of having less screens for below-average risk women?
   3. How would you feel about coming to screening less often?
4. How would you feel if a HCP told you that your screening has changed and you will be seen for a screen in 6 years time? (prompt: explore different types of HCP, e.g. doctor, mammographer).
   1. Would you be reassured or concerned?
   2. How direct should HCPs be about coming to screening every 6 years instead of every 3?
   3. What would you want to know from the HCPs?
   4. Would you trust the HCPs advice to have screening every 6 years?
5. You could be told that, ‘you are at below-average risk so you will be invited to screening every 6 years’.
   1. Would you be happy about that? Why?
6. If it was a choice to come to screening every 6 years or stay at 3 years, how would you decide whether or not to opt for screening every 6 years instead of every 3?
   1. What information would you need to know to make that decision?
      1. What would be reassuring to know?
   2. Would you make that decision alone or discuss it with others (i.e. family, friends, HCPs)?
   3. What do you think are the main reasons why women who are at below-average risk would want to choose less frequent screening?
   4. What do you think are the main reasons why women who are at below-average risk would NOT want to choose less frequent screening?
7. If screening was to change for you, how would you like to receive information about coming to screening every 6 years?
   1. Leaflets, letters, face-to-face consultations?
   2. You found out your risk via letter. How appropriate would this have been if there had been a change to your screening?
   3. From whom would you like to receive this information? (Prompts: BSP, GP, Clinician?).
8. What information do you think women at below-average risk need to remain breast aware if they were coming for screening every 6 rather than every 3 years?
9. Do you feel you know enough about the harms and benefits of screening?
   1. What do you know about false positives/over diagnoses? {EXPLAIN HERE ABOUT FP/OD IF NEEDED}
   2. Does/Would knowing this information make you feel/think differently about having screening every 6 years?
10. Would you personally want screening every 6 years instead of every 3 years?
    1. What would be your reasons to have screening every 6 years (or not choosing every 6 years [dependent on answer])?
11. How do you think we could best support women like yourself who are at low risk if a change in screening was to be implemented?
    1. How do you think we could support women from different backgrounds?

**[Give women screening materials/let them read/ keep recorder on]**

**Screening materials questions**

1. Is there anything that needs to be in the letter/leaflet that isn’t?
2. As it is now would this leaflet help you make a decision about coming to screening less frequently? Why?
3. How would you feel about coming to screening every 6 years after reading this information? (Prompt: specifically about the Hs/Bs).
4. Is there anything that you think should be in the leaflet to help you make a decision about coming to screening every 6 years?
5. What information should be in there about coming to screening every 6 years?
6. Are there better ways of us delivering this information?
7. Is there anything that you think shouldn’t be in there?
8. Is there anything else that you would need?

***What do you believe is the key take home message/s for the research team about changing screening for low risk women?***

**Closing comments**

Thank you for talking to me today and sharing your views. Your insights will be invaluable when we come to pilot this change to screening for low risk women.

- Was there anything that you would like to add?
- Was there anything that you thought we’d discuss and haven’t?
- Do you have any questions for me?

The recording of this interview will be transcribed and the original recording will be stored until we have written the findings up for publication. We will take out any identifiable information so that nothing you have said can be traced back to you. If you have any questions after today you can contact us on the details provided in the information sheet. If you would like a copy of the results after all the interviews have been completed please let us know and we will send you a copy of the final report.

I’d just like to thank you again for taking part.

**Appendix 2: interview schedule (national screening figurres)**

**Introduction to topic**

Thank you for agreeing to meet me today. As you may know we are currently running a study where across 5 sites in the North West of England women are given the opportunity to find out their risk of developing breast cancer. We are able to calculate that by obtaining information on lifestyle and family history and combining that with women’s mammographic breast density. Providing women with their risk is especially important for high risk women as they could have access to increased screening, of once every 12/18 months, or they could be offered chemoprevention medication.

It has been suggested that reducing the frequency of screening that low risk women receive could be beneficial to women. And as there appear to be more harms associated with breast screening that benefits in the form or false positives and unnecessary treatments, an increased screening interval that goes beyond the current 3 year guideline could actually maximise benefits and minimise these harms.

This study is running alongside the study I have outlined. The aim of today therefore is to find out from yourself what you think about this proposal and what would be acceptable in your eyes. However we are interested in your thoughts and opinions and what you would deem acceptable. We are also interested in how this proposal would be communicated to women and what information they would need to make a decision.

I also have to mention that in the unlikely event that anything is discussed that constitutes mal-practice or misconduct I have a duty to report such a disclosure and would therefore have to break confidentiality.

Do you have any questions before we begin?

**Discussion points**

**Introduction to topic**

- What is the feasibility of introducing an increased screening interval beyond 3 years for low risk women into the NHSBSP?

**Implementation criteria**

- If this were to be implemented, what criteria should inform this change? What should we consider? (**prompts:** Financial cost of screening; Number of missed/interval cancers; Number of overdiagnosed; mortality)

Risk Threshold

- What risk threshold should be used to recommend an increased screening interval?
- Low risk women could be at different ends of the categories, i.e. some women could be at 0.5% risk of breast cancer and others at 1.5%. What do you think about this? (**Prompt:** consideration when suggesting less frequent screening?)

Interval length

- What would be an acceptable length between screening intervals?
  - When should it be introduced? (**prompt:** age of 1st screen)
  - When should risk be re-evaluated?

Implementation

- How do you think a screening programme like this should be implemented? / How do you see this working within the NHS BSP?

Information provision

- What information do you think women would need in order to make a decision about making a change in their screening? **/** What should women know about why an increased screening interval is being recommended? (**prompt**: cost of high risk screening; ‘safety’ /Harms of screening)
- How should women receive information about this proposal? **Prompts:**
  - Via a letter or leaflet?
  - Face to face?

Informed decision-making

- What is the best way to facilitate informed choice? (**prompt:** own decision; HCP advice; PHE advice; both)
- How can women be best supported, especially women from BAMER backgrounds and women of low SES.
- What advice should healthcare professionals give?
- How should women opt for increasing their interval between screenings? (**prompt:** Opt in or opt out)
  - Should this be open to change?
  - How could women be able to change it?

**Implications of risk-stratified screening**

- What are the benefits to low risk women if they were to opt for an increased screening interval?
- What service implications do you foresee with this proposal? (**prompt:** capacity/workload; financial’ high risk women; less overdiagnosis?)
- What issues could women face (and how could they be mitigated)?
- What issues could healthcare professionals encounter (and how could they be mitigated)?
- What issues could policy makers encounter (and how could they be mitigated)? (**Prompt:** media, lobby groups, trust, credibility)
- What do you think is the most important consideration for implementing risk stratified screening? (**prompt:** screening interval; risk threshold; age at which women start)

***Note- always explore natural areas of important discussion.***

**Closing comments**

Thank you again for meeting me today and sharing your views. Your insights and discussions will be invaluable when we could to pilot this initiative.

- Was there anything that you would like to add? Any questions?
- Was there anything that you thought we would discuss and haven’t?

**Appendix 3: interview schedule (healthcare professionals)**

*For ethics committee and researchers: this study is about exploring the views of healthcare professionals (HCPs) who work in the breast screening programme with regards the proposal to increase the screening interval for women who are identified as low risk of developing breast cancer. The focus group/interview will focus on, (1) how feasible is it to introduce risk stratified screening for low risk women into the breast screening programme, (2) what should healthcare professionals be advising women and what do they feel comfortable with advising, (3) what information will women need to make a decision about reducing the frequency at which they attend for screening, (4) how should women receive information about increasing the screening interval beyond 3 years and reducing the frequency at which they attend for screening and (5) how should women be supported, especially women of low SES and women from BAME groups. Throughout, the focus group/interview will focus on what is acceptable for women and the role healthcare professionals should play in advising women about an increased screening interval.*

**Introduction to topic**

Researcher:

The focus group/interview could take up to an hour and a half depending on how much you have to say and contribute. We can stop the focus group/interview at any point if you feel we need to and you can leave at any point you wish. As stated in the PIS, this session will be recorded so that we can concentrate on what you are saying in the moment and accurately present your data later on. Any data collected from you will be totally anonymised and confidential. I may ask you at times to revisit a point or clarify what you mean in order to truly understand what you are saying and discussing. I also ask you to respect the privacy of others and allow others the time to discuss their views. I also have to mention that in the unlikely event that anything is discussed that constitutes mal-practice or misconduct I have a duty to report such a disclosure and would therefore have to break confidentiality.

P.I/Study Representative:

Thank you for agreeing to take part in the study. As you may know we are currently running a study where across 5 sites in the North West of England women are given the opportunity to find out their risk of developing breast cancer. We are able to calculate that by obtaining information on lifestyle and family history and combining that with women’s mammographic breast density. Providing women with their risk is especially important for high risk women as they could have access to increased screening of once every 12/18 months, or they could be offered chemoprevention medication, such as Tamoxifen and Raloxifene.

It has been suggested that reducing the frequency of screening that low risk women receive could be beneficial. And as there appear to be more harms associated with breast screening that benefits in the form of false positives and unnecessary treatments, an increased screening interval above 3 years could actually maximise benefits and minimise these harms.

This study is running alongside the study I have outlined. The aim of today therefore is to find out from yourselves/yourself what you think about this proposal and what would be acceptable in your eyes. So for example we have set a risk level of 1.5% and 0.5% for oestrogen-negative breast cancer as an acceptable threshold for the increased screening interval option. However we are interested in your thoughts and opinions and what you would deem acceptable. We are also interested in how this proposal should be communicated to women and what information they would need to make a decision. As well as the acceptability to women we are interested in how you as HCPs working in the screening programme will be affected and what you would feel comfortable with.

Do you have any questions before we begin?

**Discussion points**

These discussion points are related to the aims of the interviews:

- Does anyone have any general views about what has just been discussed?
- How would you like to see this implemented if an increased screening interval for low risk women was introduced?
- What is the feasibility of introducing an increased screening interval beyond 3 years for low risk women into the NHSBSP?
- What factors should inform an increased screening interval for low risk women? Cost of screening? Number of missed cancers? Number of overdiagnosis? Risk Threshold.
- What risk threshold should be used to recommend an increased screening interval?
- As a HCP who works in the screening service what threshold of risk would you be comfortable with discussing with a woman who may be eligible for an increased screening interval? (Prompt: For example you could have women at two extremes of the category, i.e. at 0.5% and 1.5%.) Context – if a women were to ask what you think etc.
- What would be an acceptable length between screening intervals?
  - When should it be introduced (prompt: age of 1^st^ screen)
  - When should risk be re-assessed?
- It is hoped that stratified screening for low risk women will cost less and screening for high risk women will cost more. How do you feel about that? How comfortable are you with this? What financial implications do you foresee with the proposal of risk stratified screening?
- In thinking about implementation, what issues could you encounter? (prompt: Complaints; Queries; Who is the contact for questions? How could these be mitigated?
- What information will women need to make a decision about their screening and in what form should they receive this information?
- As HCPs working in the NHSBSP what advice would you be comfortable in giving women? Who should be giving the advice?
- What should women know about why reducing the frequency at which they attend for screening is being recommended, i.e. because of the cost of high risk screening? Harms of screening?
- How should the information be communicated?
  - Via letter?
  - Face to face?
- How would women opt for this change in their screening?
  - Opt in or opt out option?
- What are benefits of having an increased screening interval for low risk women?
- What are the potential issues associated with an increased screening interval for low risk women? How could these be mitigated?
- What psychological impact could reducing the frequency at which women attend for screening have on low risk women? With these factors in mind how comfortable would you feel in discussing this change with women?
- What is the best way to facilitate informed choice? (prompt: own decision; HCP advice; PHE advice; both)
- Do you foresee this change biasing choice? Prompt – Effecting uptake?
- How can different groups of women be supported? SES, BAMER?
- What do you think is the most important consideration for implementing risk stratified screening for low risk women?

***Note- always explore natural areas of important discussion***

***Summary of ideas.***

**Closing comments**

Thank you again for meeting me today. Your insights and discussions will be invaluable when we come to pilot this initiative.

- Was there anything that you would like to add?
- Was there anything that you thought we would discuss and haven’t?
- Do you have any questions for me?

The recording of this focus group/interview will now be transcribed. We will take out any identifiable information so that nothing you have said can be traced back to you. If you have any questions after today you can contact us on the details provided in the information sheet. If you would like a copy of the results please let us know and we will send them out to you. Thank you again for taking part.
